# Reaction of Winter Cover Crops to *Meloidogyne enterolobii* and Glasshouse Bioassay for Evaluating Utility in Managing *M. enterolobii* in Soybeans

**DOI:** 10.2478/jofnem-2023-0014

**Published:** 2023-05-02

**Authors:** Neel Saha, Tanner Schwarz, Samantha Mowery, Adrienne M. Gorny

**Affiliations:** Department of Entomology and Plant Pathology, North Carolina State University, Raleigh, NC 27695, USA

**Keywords:** biomass, Guava root-knot nematode, interaction, management, reproductive factor

## Abstract

*Meloidogyne enterolobii* is an invasive and highly aggressive root-knot nematode pathogen impacting the Southeastern United States. Winter cover cropping may be a cost-effective method for reducing populations of *M. enterolobii* in between summer cash crops, yet a gap in the knowledge remains about the response of these cover crops to *M. enterolobii* and their utility in suppressing nematode populations prior to a cash crop. A “two-step” glasshouse bioassay was performed to evaluate eight winter cover crops popular in North Carolina for their direct response to *M. enterolobii* infection, and to quantify their effect in reducing nematode populations for the following soybean plants. Data on cover crop root galling, soybean root galling, soybean shoot fresh weight, soybean root fresh weight, eggs per gram of soybean root, and a modified reproductive factor were collected. Cereal cover crops did not display root galling, and there was significantly less root galling in those soybean plants following cereal winter cover crops when compared to those following broadleaf winter cover crops. Broadleaf winter cover crops resulted in significantly higher eggs per gram of soybean root and modified reproductive factor in the soybean plants, compared to cereal cover crops and non-inoculated controls. Results from this study suggest that cereal winter cover crops may be poor-hosts to *M. enterolobii* and may significantly reduce *M. enterolobii* populations before a soybean crop, compared to broadleaf winter cover crops. This study lays the groundwork for management recommendations and future field trials to assess management of *M. enterolobii* through winter cover cropping.

The plant-parasitic nematode *Meloidogyne enterolobii* Yang and Eisenback, 1983 (syn. *M. mayaguensis*) is an invasive species to the Southeastern region of the contiguous United States (U.S.), having first been reported in Florida in 2001, and subsequently in North Carolina and South Carolina (Brito et al., 2004; Ye et al., 2013; Rutter et al., 2019). The species has been described as highly virulent, even reproducing on crop cultivars possessing root-knot nematode resistance genes such as tomato (*Mi-1* gene), soybean (*Mir1* gene), and bell pepper (*N* gene) (Williamson and Roberts, 2009; Castagnone-Sereno, 2012). Because of the lack of genetic host resistance available in commercial crop varieties, the current management options in the Southeastern U.S. rely heavily on cultural and chemical tactics, including the use of fumigant and non-fumigant nematicides, rotation to non-host crops, and sanitation measures to prevent further spread of the nematode. The use of non-host cover crops has recently gained attention by many producers, agricultural extension personnel, and crop consultants, as it offers a possible economically viable alternative to costly fumigants. Further, the use of cover crops provides pathogen management options for organic production or low input systems. Yet the utility and impact of cultural tactics such as cover cropping for suppression of *M. enterolobii* populations remains understudied.

Cover crops are grown between cash crop cycles or incorporated within cash crop cycles to cover land that is not utilized for primary production. Cover cropping has been implemented to improve soil structure, reduce soil erosion, increase organic matter, increase available nitrogen, suppress weed populations, and suppress pathogens and pests like nematodes within the context of various cropping systems (Sainju and Singh, 1997; Lu et al., 2000; Hartwig and Ammon, 2002). The use of cover crops for plant-parasitic nematode suppression mainly focuses on the host status of the cover crop, yet certain cover crops (e.g., brassica species and some sudangrasses) may also be leveraged for their biofumigant properties as “green manures” (Kruger et al., 2013). Non-host or poor-host cover crops serve to reduce nematode populations and reproduction between cash crop cycles, resulting in lower initial nematode populations and reduced pathogen pressure on the main cash crop.

Previous work has reported the host status of several summer cover crop plant species to *Meloidogyne enterolobii*. Sunn hemp (*Crotalaria juncea*) and sorghum-Sudangrass (*Sorghum biocolor* x *S. sudanense*) have been identified as non-host to *M. enterolobii* (Bui and Desaeger, 2021; de Brida et al., 2018). Reported host summer cover crop species include sunflower (*Helianthus annuus*) and cowpea (*Vigna unguiculata*) (Bui and Desaeger, 2021). Further, Brito et al. (2007) reported the mustard ‘Florida Broad Leaf’ (*Brassica oleracae*) to be a good host to *M. enterolobii*. Khanal and Harshman (2022) corroborated sunflower and cowpea as host and sunn hemp and sorghum sudangrass as non-host to *M. enterolobii*. They further reported that buckwheat (*Fagopyrum esculentum*) and sesame (*Sesamum indicum*) were poor-host, while pearl millet (*Pennisetum glaucum*) and grain sorghum (*Sorghum bicolor*) were non-host (Khanal and Harshman, 2022).

In contrast to summer cover crops, winter cover crops are grown in rotation with summer cash crops. Winter cover crops are generally planted during the fall after harvest of the main cash crops, allowed to grow over the winter months, and are then either harvested or terminated in early spring before the planting of another main cash crop. Winter cover crops serve to utilize residual NO_3_, with some species of winter cover crop serving to increase the organic matter content in the soil and, particularly with leguminous species, enhancing available nitrogen (Sainju and Singh, 1997). Winter cover crops are generally species that can survive cooler and cold temperatures, including cereals such as oat, wheat, rye, and barley, as well as leguminous species such as crimson clover, winter pea, and some vetches. Several species of brassica, including mustard and canola (rapeseed), are also utilized as winter cover crops by some farmers. In the Southeastern U.S., oats (*Avena sativa*) and wheat (*Triticum aestivum*) are commonly used as winter cover crops, and these have been demonstrated to be non-host to *M. enterolobii* (de Brida et al., 2018).

Although several summer cover crops have been identified as host or non-host to *M. enterolobii*, the response of winter cover crops to *M. enterolobii* remains less explored. Further, a gap remains in the understanding of how the use of a winter cover crop may translate into reducing *M. enterolobii* initial populations for a following cash crop. The objectives of this study were (1) to evaluate winter cover crops common in agricultural systems of the southeastern U.S. for their response to *Meloidogyne enterolobii* and (2) to quantify through a greenhouse bioassay the utility of these winter cover crops in reducing initial populations of the nematode for a successive soybean crop. This study was intended to inform future field-based experiments to explore the impact of winter cover crops on *M. enterolobii* management in soybean under field conditions.

## Materials and Methods

*Nematode culture and extraction:* A culture of *Meloidogyne enterolobii* was established from infected soil collected from a soybean field in Wilson County, North Carolina in 2017, and has been continuously maintained on tomato plants (cv. ‘Rutgers’) in the glasshouse at North Carolina State University. Identity of this isolate is periodically checked using species-specific amplification primer sets MeF/MeR (Long et al., 2006) and Ment17F/Ment17R (Kiewnick et al., 2015). The potting media used for maintaining cultures was a steam-sterilized 1:1 soil-to-sand mixture. To extract *M. enterolobii* eggs for inoculating cover crop plants, tomato culture plant root systems were destructively harvested and rinsed in tap water to remove excess soil, then soaked in a 10% household bleach solution (6% available NaOCl) for 45 sec to break down the gelatinous matrix surrounding mature egg sacs, aided by hand through gentle agitation (Hussey and Barker, 1973). Nematode eggs released from the culture plant root system were recovered by passing the extraction solution through stacked 250 μm, 75 μm, and 25 µm mesh sieves and rinsing with fresh tap water to remove residual NaOCl. Eggs were collected from the 25 μm mesh sieve in a final volume of 35 ml. To remove additional soil and plant tissue debris, 15 ml of a 2.05 M sucrose solution was added to each sample, gently inverted, then centrifuged at 1,000 rcf for 5 min. Nematode eggs suspended in the sucrose solution were recovered by passing the supernatant through a 25 µm mesh sieve, rinsing with fresh tap water, collecting, and suspending in tap water. Extracted egg samples were counted under a compound inverted microscope (Nikon Instruments Inc., Melville, NY, USA) at 100x magnification. Extracted eggs were stored at 4°C for 24 h prior to inoculation.

*Plant growth, inoculation, and assessment:* Seeds of eight different winter cover crop species were obtained (Johnny’s Select Seeds, Winslow, ME, USA) (Table 1). Seeds (approx. 1 g per pot) were sewn into 15.2 cm diameter clay pots filled with steam-sterilized 1:1 soil-to-sand mix. Pots were amended with slow-release fertilizer (Osmocote Smart-Release Plant Food, The Scotts Company, Marysville, OH, USA) and watered daily. Three weeks after sowing, pots were inoculated with 10,000 *M. enterolobii* eggs per pot by creating three small holes, approx. 0.5 cm wide by 3 cm deep equidistant from each other and pipetting inoculum into the holes. Eight pots of each cover crop species were included, with each pot considered as a replicate. Non-inoculated pots of crimson clover (*Trifolium incarnatum*) and ryegrass (*Lolium multiflorum*) were included as non-inoculated control. Cover crops were maintained in the glasshouse for 60 days post inoculation at 26– 28°C with no supplemental lighting. At 60 days post inoculation, cover crop plants were destructively harvested and the presence or absence of root galling was recorded, whereas presence was defined as root galling severity of ≥ 1 on a severity index scale of 0–10 (Bridge and Page, 1980). From each pot, 200 g of soil was removed and transferred to a new 15.2 cm diameter clay pot; cover crops’ roots were discarded after root galling evaluation and were not transferred with the soil. These new pots were topped with additional, fresh soil mixture (steam-sterilized 1:1 soil-to-sand mixture) and three soybean seeds (*Glycine max*; variety not stated; Sustain Seed+Soil, Minster, OH, USA) were seeded into each pot. Soybean plants were germinated, thinned to one plant per pot, and maintained in the greenhouse at 26–28°C with no supplemental lighting. At 60 days post germination, soybean plants were destructively harvested. Plants were gently removed from the pots, preserving integrity of the root system, and roots rinsed with tap water to remove excess soil. Soybean fresh shoot biomass and fresh root biomass were measured by cutting the plants at the crown and weighing foliage and roots separately. Soybean root galling severity was estimated on a scale from 0–100% using a scale modified from Bridge and Page (1980). Nematode eggs were then extracted from soybean roots following the procedure described above and counted under a compound inverted microscope. The total number of eggs per plant was estimated by calculating the total eggs per ml of extraction solution, then multiplying by the total volume of extraction solution (50 mL). The number of eggs per gram of soybean root tissue was calculated by dividing the total number of eggs in each sample by the weight of the soybean fresh root system. A modified reproductive factor (RF_mod_) was calculated for nematode populations on each soybean plant by dividing the total number of eggs extracted from the soybean plant (final population; P_f_) by the initial number of eggs inoculated to the preceding cover crop (initial population; P_i_, *n* = 10,000). The experiment was performed twice (with Trial 1 being performed from April to September 2021, and Trial 2 being performed from November 2021 to April 2022).

**Table 1.** Host status of winter cover crops and response of soybean after inoculation of the preceding cover crop with the root-knot nematode *Meloidogyne enterolobii,* Trial 1. Means (*n* = 8) followed by the same letter within each column are not significantly different at the 0.05 level.

Plant species	Common name	Root galling in cover crop^a^	Soybean shoot weight (g)	Soybean root weight (g)	Soybean root galling severity
*Hordeum vulgare*	Barley	−	41.4 bc	52.0 abc	0 c
*Trifolium incarnatum*	Crimson clover	+	24.8 d	38.8 c	59.4 ab
*Guillenia flavescens*	Yellow mustard	+	31.2 cd	35.9 c	48.1 b
*Avena nuda*	Streaker hulless oats	−	42.6 bc	51.1 abc	0 c
*Lolium multiflorum*	Ryegrass	−	50.8 b	57.2 ab	0 c
*Secale cereale*	Winter rye	−	53.0 ab	49.9 abc	0 c
*Vinca villosa*	Hairy vetch	+	26.4 cd	51.4 abc	67.9 a
*Triticum aestivum*	Spring wheat	−	43.3 bc	44.1 bc	0 c
*Lolium multiflorum*	Ryegrass (non-inoculated)	−	65.9 a	67.0 a	0 c
*Trifolium incarnatum*	Crimson clover (non-inoculated)	−	65.6 a	63.7 a	0 c
*P-value* ^b^			<0.0001	0.0369	<0.0001
MSE^c^			166.4	295.7	169.2

a Presence or absence of root galling was evaluated whereas presence was defined as root galling severity of ≥1 on a severity index scale of 0–10 (Bridge and Page 1980). ‘-’ indicates absence of galling, ‘+’ indicates presence of galling.

b Significance value of Fisher’s least significant difference.

c Mean squared error.

*Statistical analysis:* Data sets from Trial 1 and Trial 2 were assessed to determine if they could be combined by comparing means using a Wilcoxon rank sum test (Mann and Whitney, 1947) and evaluating homogeneity of variances using Fisher’s F-Test (Markowski and Markowski, 1990). Evidence of variability was observed; therefore, the data sets from each trial were analyzed separately. The main effects of the preceding winter cover crop on soybean fresh shoot and root biomass, soybean root galling severity, the number of eggs per gram of soybean root, and RF_mod_ were evaluated using analysis of variance within RStudio (v. 3.6.2; R Core Team, 2019) with replicate as a random effect, and removing a single outlier with high leverage in Trial 1. For each trial dataset, the assumptions of normality and homogeneity of variances in the data were verified by visualization of the data and performing the Shapio-Wilks (function ‘shapio.test’) and Levene’s (function ‘leveneTest’ within the ‘car’ package) tests. Where significant differences were identified, Fisher’s Least Significant Difference test was used to separate means (function ‘LSD.test’ within the ‘agricolae’ package).

## Results

**Figure 1:**
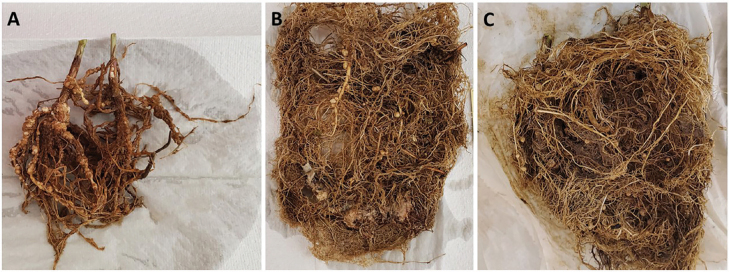
Effect of preceding winter cover crop on soybean roots following (A) yellow mustard, (B) barley, and (C) ryegrass. Significant root galling was observed in soybean roots following yellow mustard, crimson clover, and hairy vetch, while no root galling was observed on the roots of barley, Streaker hulless oats, ryegrass, winter rye, or spring wheat.

*Winter cover crops as host to* *Meloidogyne enterolobii:* In both Trial 1 and Trial 2, root galling symptoms typical of infection by *Meloidogyne enterolobii* were observed on roots of crimson clover (*Trifolium incarnatum*), yellow mustard (*Guillenia flavenscens*), and hairy vetch (*Vinca villosa*). No root galling was observed on the roots of barley (*Hordeum vulgare*), Streaker hulless oats (*Avena nuda*), ryegrass (*Lolium multiflorum*), winter rye (*Secale cereale*), or spring wheat (*Triticum aestivum*) (Tables 1,3; Figure 1). No root galling was observed in the non-inoculated control plants in either trial (Tables 1,3).

**Table 3.** Host status of winter cover crops and response of soybean after inoculation of the preceding cover crop with the root-knot nematode *Meloidogyne enterolobii,* Trial 2. Means (*n* = 8) followed by the same letter within each column are not significantly different at the 0.05 level.

Plant species	Common name	Root galling in cover crop^a^	Soybean shoot weight (g)	Soybean root weight (g)	Soybean root galling severity
*Hordeum vulgare*	Barley	−	14.92	31.59	0.3 d
*Trifolium incarnatum*	Crimson clover	+	10.09	16.20	26.7 b
*Guillenia flavescens*	Yellow mustard	+	10.09	18.83	14.6 c
*Avena nuda*	Streaker hulless oats	−	11.39	20.32	0 d
*Lolium multiflorum*	Ryegrass	−	6.35	17.71	0.3 d
*Secale cereale*	Winter rye	−	12.65	22.15	0.3 d
*Vinca villosa*	Hairy vetch	+	5.03	13.58	46.2 a
*Triticum aestivum*	Spring wheat	−	8.31	20.01	0.3 d
*Lolium multiflorum*	Ryegrass (non-inoculated)	−	11.99	20.12	0 d
*Trifolium incarnatum*	Crimson clover (non-inoculated)	−	11.47	17.41	0 d
*P*-value^b^			0.070 (ns)	0.063 (ns)	<0.0001
MSE^c^			33.1	88.0	121.5

a Presence or absence of root galling was evaluated whereas presence was defined as root galling severity of ≥ 1 on a severity index scale of 0–10 (Bridge and Page 1980). ‘-’ indicates absence of galling, ‘+’ indicates presence of galling.

b Significance value of Fisher’s least significant difference

c Mean squared error

*Response of soybean to inoculation of preceding winter cover crop:* In Trial 1, there were significant differences in soybean shoot fresh weight among treatment groups of preceding winter cover crops, with the non-inoculated control treatments having the greatest soybean shoot fresh weight, and crimson clover having the lowest soybean shoot weight (Table 1). In Trial 2, there were no significant differences in soybean shoot weight among treatment groups (Table 3). In Trial 1, there were significant differences in soybean root fresh weight among treatment groups, with crimson clover, yellow mustard, and spring wheat having significantly lower soybean root weights than the non-inoculated control plants (Table 1). In Trial 2, there were no significant differences in soybean root fresh weight among treatment groups (Table 3). In Trial 1, there were significant differences in soybean root galling severity, with crimson clover, yellow mustard, and hairy vetch having significantly greater severity of root galling than all other treatment groups (Table 1). In Trial 2, an identical trend was observed, with crimson clover, yellow mustard, and hairy vetch having significantly greater severity of root galling than all other treatment groups (Table 3).

*Nematode reproduction on soybean plants after inoculation of preceding winter cover crop:* In Trial 1, there were significant differences in the number of eggs per gram of soybean root, with soybean plants following crimson clover, yellow mustard, and hairy vetch having significantly more eggs per gram of root than all other treatment groups (Table 2). An identical trend was observed in Trial 2, with soybean plants following crimson clover, yellow mustard, and hairy vetch having significantly more eggs per gram of root than all other treatment groups (Table 4). In both Trial 1 and Trial 2, there were significant differences in RF_mod_, with soybean plants following crimson clover, yellow mustard, and hairy vetch having a significantly higher RF_mod_ than all other treatment groups (Tables 2,4). There was a low level of eggs per gram of soybean root in non-inoculated controls (Table 2,4).

**Table 2.** Reproduction of *Meloidogyne enterolobii* on soybean after inoculation of preceding winter cover crop plants, Trial 1. Means (*n* = 8) followed by the same letter within each column are not significantly different at the 0.05 level.

Plant species	Common name	Eggs per gram of soybean root tissue	RF _mod_^a^
*Hordeum vulgare*	Barley	10.1 b	0.05 b
*Trifolium incarnatum*	Crimson clover	1914.2 a	7.84 a
*Guillenia flavescens*	Yellow mustard	3104.3 a	7.19 a
*Avena nuda*	Streaker hulless oats	15.2 b	0.07 b
*Lolium multiflorum*	Ryegrass	6.8 b	0.03 b
*Secale cereale*	Winter rye	6.9 b	0.04 b
*Vinca villosa*	Hairy vetch	2195.3 a	11.33 a
*Triticum aestivum*	Spring wheat	6.5 b	0.03 b
*Lolium multiflorum*	Ryegrass (non-inoculated)	3.5 b	0.02 b
*Trifolium incarnatum*	Crimson clover (non-inoculated)	4.3 b	0.02 b
*P-value* ^b^		0.0001	< 0.0001
MSE^c^		2380380	19.5

a Modified Reproductive Factor, calculated by dividing the total number of eggs extracted from soybean root systems (final population), by the initial number of eggs inoculated to the preceding cover crop (initial population; herein, 10,000).

b Significance value of Fisher’s least significant difference.

c Mean squared error.

**Table 4.** Reproduction of *Meloidogyne enterolobii* on soybean after inoculation of different proceeding winter cover crop plants, Trial 2. Means (*n* = 8) followed by the same letter within each column are not significantly different at the 0.05 level.

Plant species	Common name	Eggs per gram of soybean root tissue	RF _mod_^a^
*Hordeum vulgare*	Barley	13.7 c	0.06 c
*Trifolium incarnatum*	Crimson clover	6041.5 a	7.13 a
*Guillenia flavescens*	Yellow mustard	1452.4 bc	3.71 b
*Avena nuda*	Streaker hulless oats	3.7 c	0.01 c
*Lolium multiflorum*	Ryegrass	10.8 c	0.02 c
*Secale cereale*	Winter rye	5.0 c	0.01 c
*Vinca villosa*	Hairy vetch	2476.6 b	2.15 bc
*Triticum aestivum*	Spring wheat	9.2 c	0.02 c
*Lolium multiflorum*	Ryegrass (non-inoculated)	16.4 c	0.02 c
*Trifolium incarnatum*	Crimson clover (non-inoculated)	0 c	0 c
*P*-value^b^		< 0.0001	0.0003
MSE^c^		2396198	11.1

a Modified Reproductive Factor, calculated by dividing the total number of eggs extracted from soy bean root systems (final population), by the initial number of eggs inoculated to the preceding cover crop (Initial population; herein, 10,000).

b Significance value of Fisher’s least significant difference.

c Mean squared error.

## Discussion

*Meloidogyne enterolobii* is an emerging nematode pathogen in the Southeastern U.S., where it poses the risk of significantly affecting agricultural production. Soybean (*Glycine max*) is susceptible to *M. enterolobii*, and the nematode species is able to overcome and infect even those cultivars possessing the *Mir1* root-knot nematode resistance gene (Castagnone-Sereno, 2012). In the authors’ experience, growers’ awareness of nematode pathogens and the interest in using cultural pathogen management tactics (such as winter cover cropping) has increased in North Carolina in recent years. However, gaps in knowledge remain, specifically regarding the utility of winter cover crops in reducing *M. enterolobii* populations in between cash crops. The objectives of the present study were to evaluate several winter cover crops common in North Carolina for their response to *M. enterolobii*, and to quantify through a glasshouse bioassay the suppressive effect of these winter cover crops on *M. enterolobii* initial populations to a following soybean crop.

In this study, all of the cereal winter cover crops assessed, including barley, Streaker hulless oats, ryegrass, winter rye, and spring wheat, did not produce galling symptoms (all less than 0.3 severity across both trials). Furthermore, the eggs per gram of soybean root and RF_mod_ were also significantly lower following cereal cover crops than the three broadleaf cover crops assessed. The broadleaf cover crops assessed, including yellow mustard, hairy vetch, and crimson clover, all produced significantly more severe soybean root galling, eggs per gram of soybean root, and increased RF_mod_. In addition, the broadleaf cover crops had directly observable root galling. This response supports the conclusion that the broadleaf winter cover crops evaluated here may be good-host to *M. enterolobii*, while the cereal winter cover crop species may be poor-host to *M. enterolobii*. In both Trial 1 and Trial 2, there was a small number of eggs recovered from the roots of soybean plants from non-inoculated control cover crop plants. This may have been due to splash dispersal of infested soil from neighboring inoculated pots, or may have been the result of possible contamination of shared extraction tools such as sieves during the harvesting and evaluation step of the experiments. Due to this result in the control plants, we refrain from concluding that any of the cover crop species tested are complete or strong non-host to *M. enterolobii*, but rather conclude that they are likely poor-host to the nematode.

De Brida et al. (2018) reported that several cultivars of wheat (*Triticum aestivum*), oat (*Avena sativa*), and sorghum (*Sorghum bicolor*) were non-host to *M. enterolobii*, and reported a significantly lower galling index, egg mass index, and reproductive factor compared to a tomato control. Bui and Desaeger (2021) evaluated several summer cover crops for their host status to *M. enterolobii*. They found that sunn hemp (*Crotalaria juncea*) and sorghum-Sudangrass (*Sorghum bicolor x S. sudanense*) were poor-host to the nematode, while sunflower (*Helianthus annuus*) and cowpea (*Vigna unguiculata*) were good-host (Bui and Desaeger, 2021). More recently, Khanal and Harshman (2022) also performed an experiment in which the host statuses of several summer cover crops were evaluated to *M. enterolobii*. They reported that warm-season grass cover crops, including three types of millet, sorghum, and sorghum-Sudangrass. were non-host to the nematode (Khanal and Harshman, 2022). Similarly to the results of this study, they identified broadleaf cover crops that were host to the nematode, including sunflower, cowpea, and buckwheat (*Fagopyrum esculentum*) (Khanal and Harshman, 2022). Cetintas et al. (2007) found common vetch (*Vinca sativa*) to be a host to *M. enterolobii*, consistent with the finding in this study that the related species hairy vetch (*Vinca villosa*) resulted in a high degree of root-galling due to *M. enterolobii*. The results of these previous studies taken together with the results of the present study strongly suggest that cereal crops are poor-to non-host to *M. enterolobii* and producers may consider them as a good rotational or cover crop choice for fields impacted by *M. enterolobii*, while broadleaf cover crops may support *M. enterolobii* reproduction and exacerbate field infestation.

In Trial 1 and Trial 2, a similar trend in the results was observed in the response variables related to soybean growth among the treatment groups consisting of the preceding cover crop. For example, in both trials, soybeans following hairy vetch had the highest average root galling severity score, followed by those of crimson clover and then yellow mustard (Tables 1,2). Of the cereal winter cover crops, no galling was observed in Trial 1, and only a minute degree of galling was observed in Trial 2. In contrast, a higher degree of variability was observed in response variables related to nematode reproduction. For example, there was no consistent trend between trials when considering RF_mod_ among treatment groups. Overall, soybean plant growth and nematode reproduction across all treatment groups was numerically lower in Trial 2 when compared to Trial 1. This may be because Trial 2 was performed during the winter months in North Carolina.

Glasshouse bioassays remain an important tool for nematologists, and bioassays have been used to evaluate treatments such as nematicides (e.g., de Oliveira Silva et al., 2019), or to assess crop genotypic response or pathogen virulence (e.g., Thies, 2011; Hallmann and Kiewnick, 2018), or in diagnostic capacities (e.g., Gugino et al., 2008; Gorny et al., 2021). Bioassays that use sensitive indicator crops may have a lower limit of detection compared to direct extraction, due to second-stage juveniles present infecting the plant promptly, and additional time in the assay for eggs to hatch and infect (Gugino et al., 2008; Duncan, 1991). Thus, bioassay results may support robust management decisions. Here, we demonstrate the effectiveness of a “two-step” bioassay for evaluating both the direct response of the cover crop and its ability to support or suppress nematode populations that may impact a following cash crop. However, there remain certain shortcomings of the glasshouse bioassay. For example, the temperature at which the cover crops were grown and the length of time for which they were maintained may not fully replicate field conditions, and thus, field populations of *M. enterolobii* may behave and reproduce differently than what was observed in this study. Furthermore, the design of this experiment only evaluated root galling in the cover crop; a direct measurement of nematode reproduction (such as quantifying egg production) in the cover crop was not obtained. Marquez et al. (2022) noted that simply evaluating the production of root galls is not sufficient to fully determine host status. However, we believe the complimentary data of soybean growth and nematode reproduction under the soybean crop supplement our evaluation of cover crop host status. Additionally, once cover crop roots were removed from the soil to evaluate root galling, these roots were not placed back into the soil that was then transferred to a new pot for soybean growth. This may have effectively removed a portion of the nematode population contained within egg masses attached to the cover crop roots. This is in contrast to a field setting, where winter cover crop roots would likely be incorporated through tillage and any egg masses on the roots would remain in the soil. A broad purview should be maintained, and evaluating these winter cover crop species under field conditions is necessary to support management recommendations.

Prior to this work, the response of several winter cover crops to *M. enterolobii* was not known. To the best of our knowledge, this is the first data to begin evaluating the reaction of the winter cover crops barley, crimson clover, yellow mustard, Streaker hulless oats, ryegrass, winter rye, spring wheat, and hairy vetch to *M. enterolobii*. In summary, this study identified several winter cover crop species with little galling in response to *M. enterolobii* and provided a quantitative estimate of the suppressive effect of these winter cover crops on *M. enterolobii* populations in a following soybean crop. This study will provide preliminary management recommendations for agricultural producers for managing this nematode with winter cover cropping and will also provide direction for future field studies on managing *M. enterolobii* with winter cover cropping.
